# Active transcription without histone modifications

**DOI:** 10.18632/oncotarget.6437

**Published:** 2015-11-30

**Authors:** Sílvia Pérez-Lluch, Roderic Guigó, Montserrat Corominas

**Affiliations:** Universitat de Barcelona and Institut de Biomedicina de la Universitat de Barcelona, Barcelona, Catalonia, Spain

**Keywords:** Chromosome Section, epigenetics, transcription, Drosophila, development

Gene expression is regulated by proteins such as transcription factors, as well as by chromatin modifications on DNA and histones. Some histone modifications have been associated to transcriptional activation (i.e. H3K4me1, H3K4me3, H3K9ac, H3K27ac or H3K36me3) whereas others with gene silencing (H3K9me3 and H3K27me3). However, and challenging this premise, we have identified a set of genes in *Drosophila melanogaster* that are actively expressed in the absence of the canonical histone marks [1].

By using previously published data generated by the modENCODE project [2, 3], we defined two gene sets according to their transcriptional profiles: stable genes, expressed with minor changes throughout development, and developmentally regulated genes, expressed for a short period of time, usually only in one time point. We found that whereas stable genes display histone modifications canonically associated to gene activation, the level of histone marking of regulated genes is comparable to the background levels observed in silent genes.

To discard the possibility that these observations arose from limited detection capability when monitoring histone modifications in the whole organisms (as in the modENCODE project) we analyzed recently released data on tissue-specific gene expression in third-instar larva (L3). Genes widely expressed across the whole body of the larva but only at L3 showed much lower levels of histone marks associated with active transcription than genes with tissue restricted but constant expression during development. RNA experiments confirmed that regulated genes specifically expressed at L3 are actively transcribed in the absence of H3K4me3 and H3K36me3, and their detection is not the consequence of transcription at a previous time point.

A logical inference drawn from these observations is that if histone modifications associated with activation are not necessary for the expression of developmentally regulated genes, the perturbation of the H3K4 methylation should not affect their expression. Indeed, disruption of ASH2, an essential cofactor of H3K4 trimethylation [4], reduces the expression of stable genes but does not affect that of regulated genes.

If histone modifications do not appear to play a major role in the regulation of the expression of developmentally regulated genes, how is then, the expression of these genes regulated? Our analyses of the sequence of their promoter regions and of data available on a number of transcription factors obtained though ChIP-Seq by the modENCODE consortium, suggest that binding by transcription factors plays a comparatively more important role in the control of regulated than stable genes.

With all this, we propose a model in which the expression of stable genes is controlled and maintained through cell divisions by histone marks (H3K4me3, H3K9ac, etc.). Transient binding of transcription factors, in contrast, would play the leading role in the activation and de-activation of developmentally regulated genes, the expression of which is required only for a limited period of time. As the expression of these genes is not maintained along cell divisions, the epigenetic marks would be, then, dispensable.

We think that our study opens new possibilities in the analysis of the relationship between chromatin modifications and expression, and in particular, whether the model that we propose for *Drosophila* can also be extrapolated to mammalian species.
